# Textural and Thermal Properties of the Novel Fucoidan/Nano-Oxides Hybrid Materials with Cosmetic, Pharmaceutical and Environmental Potential

**DOI:** 10.3390/ijms23020805

**Published:** 2022-01-12

**Authors:** Jakub Matusiak, Urszula Maciołek, Małgorzata Kosińska-Pezda, Dariusz Sternik, Jolanta Orzeł, Elżbieta Grządka

**Affiliations:** 1 Institute of Chemical Sciences, Faculty of Chemistry, Maria Curie-Sklodowska University in Lublin, M. Curie-Sklodowska Sq. 3, 20-031 Lublin, Poland; 2 Analytical Laboratory, Institute of Chemical Sciences, Faculty of Chemistry, Maria Curie-Sklodowska University in Lublin, M. Curie-Sklodowska Sq. 3, 20-031 Lublin, Poland; urszula.maciolek@mail.umcs.pl; 3 Department of Inorganic and Analytical Chemistry, Faculty of Chemistry, Rzeszow University of Technology, 35-959 Rzeszow, Poland; m.kosinska@prz.edu.pl; 4 Department of Physical Chemistry, Institute of Chemical Sciences, Faculty of Chemistry, Maria Curie-Sklodowska University in Lublin, M. Curie-Sklodowska Sq. 3, 20-031 Lublin, Poland; dariusz.sternik@mail.umcs.pl; 5 Department of Radiochemistry and Environmental Chemistry, Institute of Chemical Sciences, Faculty of Chemistry, Maria Curie-Sklodowska University in Lublin, M. Curie-Sklodowska Sq. 3, 20-031 Lublin, Poland; jolanta.orzel@mail.umcs.pl (J.O.); elzbieta.grzadka@mail.umcs.pl (E.G.)

**Keywords:** polysaccharide, TiO_2_, Al_2_O_3_, ZnO, stability, thermal analysis

## Abstract

The main purpose of the research was to obtain and study hybrid materials based on three different nano-oxides commonly used in the cosmetic and pharmaceutical industries: Al_2_O_3_, TiO_2_, and ZnO, with the natural bioactive polysaccharide fucoidan. Since the mentioned oxides are largely utilized by industry, there is no doubt that the presented studies are important from an environmental point of view. On the basis of the textural studies (dynamic light scattering DLS, low temperature nitrogen adsorption, X-ray diffraction analysis XRD, scanning electron microscopy SEM) it was proved that the properties of the hybrid materials differ from the pure components of the system. Moreover, the advanced thermal analysis (TG-DTG-DSC) combined with the evolved gas analysis using Fourier transformed infrared spectroscopy (FTIR) and mass spectrometry were applied to describe the thermal decomposition of fucoidan, oxides and hybrid materials. It was found that the interactions between the polymer and the oxides results in the formation of the hybrid materials due to the functionalization of the nanoparticles surface, and that their thermal stability increased when compared to the pure substrates. Such findings definitely fill the literature void regarding the fucoidan based hybrid materials and help the industrial formulators in the preparation of new products.

## 1. Introduction

Marine polysaccharides are an enormous group of substances possessing extraordinary properties that are still to be exploited [1,2]. Among them, one of the most interesting are fucoidans (FD). They are algae-derived natural polysaccharides characterized by different contents of sulphate groups in the polymer backbone [3]. The variety of such compounds has found multiple applications because of their antitumor [4,5,6], antiviral [7], antioxidant [8,9] and anticoagulant [10] properties. However, the aforementioned properties depend on different factors, such as molecular weight, sulfation degree and type of the glycosidic bonds [11]. Fucoidans are characterized by heterogeneous chemical structures. They may differ in the biogenic source, degree of purity, monosaccharide composition, sulfation content, molecular mass, d-glycosidic linkage and branching sites [12,13].

The interactions between the inorganic and organic components in the aqueous systems often lead to the formation of hybrid biocomposites [14]. In such a case, novel materials, characterized by the properties different from those of the component alone, are obtained. In the case of the cosmetic and pharmaceutical industries, three natural oxides are particularly important. These are titanium(IV), zinc(II) and aluminium(III) oxides. In addition to their high adsorptive properties [15], they are also used as carriers of bioactive substances [16,17,18], drug substrates [19] and emulsion stabilizers [20]. The studies on inorganic-organic hybrid materials provide information on their physical, chemical and biological properties. In the case of the materials that can be used in vivo and in vitro, it is important to gather data on how such species behave under different conditions. The thermal analysis of the aforementioned materials shows how the obtained composites behave under high-temperature conditions and guides their future thereof.

The purpose of this research was to investigate the physicochemical and thermal properties of the three novel fucoidan/nano-oxides hybrid materials: FD/ZnO, FD/TiO_2_ and FD/Al_2_O_3_. To this matter, a thorough characterization was performed using such techniques and methods as low-temperature nitrogen adsorption/desorption (BET), elemental analysis (CHN), X-ray diffraction analysis (XRD), and photon correlation spectroscopy (DLS). The thermal properties were studied by means of thermogravimetry (TG), differential thermogravimetry (DTG), differential scanning calorimetry (DSC) combined with Fourier-transform infrared spectroscopy (FT-IR) and mass spectroscopy (MS). Moreover, the obtained materials were analysed using scanning electron microscopy (SEM).

This is worth emphasizing that there are only a few studies on the properties of the fucoidan/nano-oxide hybrid materials [21,22]. The results show that the obtained hybrid materials are characterized by different physicochemical and thermal properties in comparison to their native components. Moreover, the characteristics of native fucoidan biopolymer is considered crucial for future biological and industrial applications. Among others, thermal properties and behaviour are important material parameters. Therefore, both classic thermogravimetric and advanced thermal analysis were performed under simulated airflow to investigate the purity and stability of the newly obtained hybrid materials. Moreover, the results were compared to those obtained for the native fucoidan (FD) and pure oxide powders. The data on the thermal degradation of fucoidan are insufficient, while according to the authors’ best knowledge, there is no information in the literature on the thermal analysis of fucoidan-oxide hybrid materials. Therefore, the results of the thermal analysis in terms of the number of degradation steps and the products of the thermal degradation are described in detail below.

## 2. Results and Discussion

Based on the earlier studies, it was confirmed that fucoidan adsorbs on all of the studied nano-oxide particles. These previous findings show that the largest adsorption was observed in the case of the FD/NaCl/ZnO system and moderate in the FD/NaCl/TiO_2_, while the smallest was found in the case of FD/NaCl/Al_2_O_3_ [23,24]. Table 1 presents the comparison of hydrodynamic diameters (D_h_) of pure oxides and fucoidan-oxide hybrid materials.

It can be observed that, despite the smallest adsorption, the largest hydrodynamic diameter of the prepared materials was observed in the case of the Al_2_O_3_-FD hybrid. Several explanations can be offered to cover this phenomenon. Firstly, the previous measurements showed that the adsorption isotherm in the case of the FD/Al_2_O_3_ system increases steadily, whereas the plateau is not reached [23]. As the inner pores of the oxide are unavailable for the polymer chains, the conformation of fucoidan on the Al_2_O_3_ surface is rich in loops and tails structures. Secondly, considering that Al_2_O_3_ has the largest particle size, the formation of bigger aggregates is more likely. Considering the textural properties of the obtained hybrid materials, some significant changes can be observed (Table 2).

It can be observed that after the functionalization of the oxide surfaces with fucoidan, the specific surface area, micropore area, external surface area, as well as micropore volume decreased. This phenomenon is related to the adsorption of the polymer on the surfaces of the used oxides. Since it was previously proven that the adsorption of fucoidan happens mostly on the surface, the inner pores are blocked by the macromolecule. Thus, the decrease of the textural parameters is observed because the adsorption centres on the surface are blocked by the tails and loops of fucoidan, and the inner pores became unavailable for adsorption.

**Figure 1:**
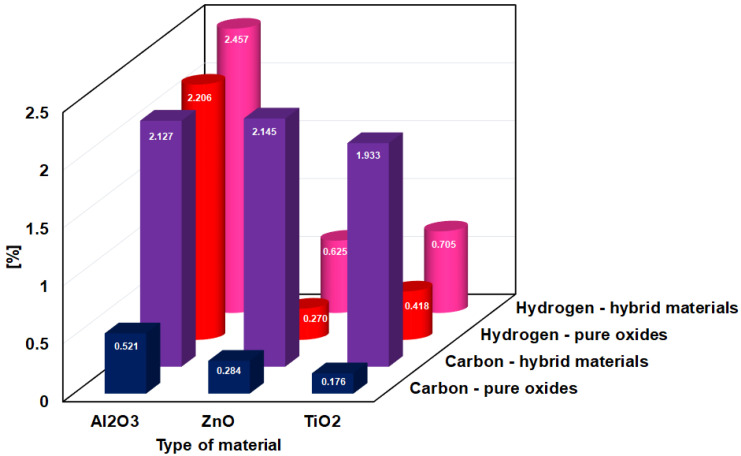
Elemental composition of the pure oxides and fucoidan-oxide hybrid materials.

The elemental composition also changes significantly. The comparison of the pure oxides and fucoidan-oxide hybrid materials shows an increase of carbon and hydrogen amounts in the samples (Figure 1).

**Figure 2:**
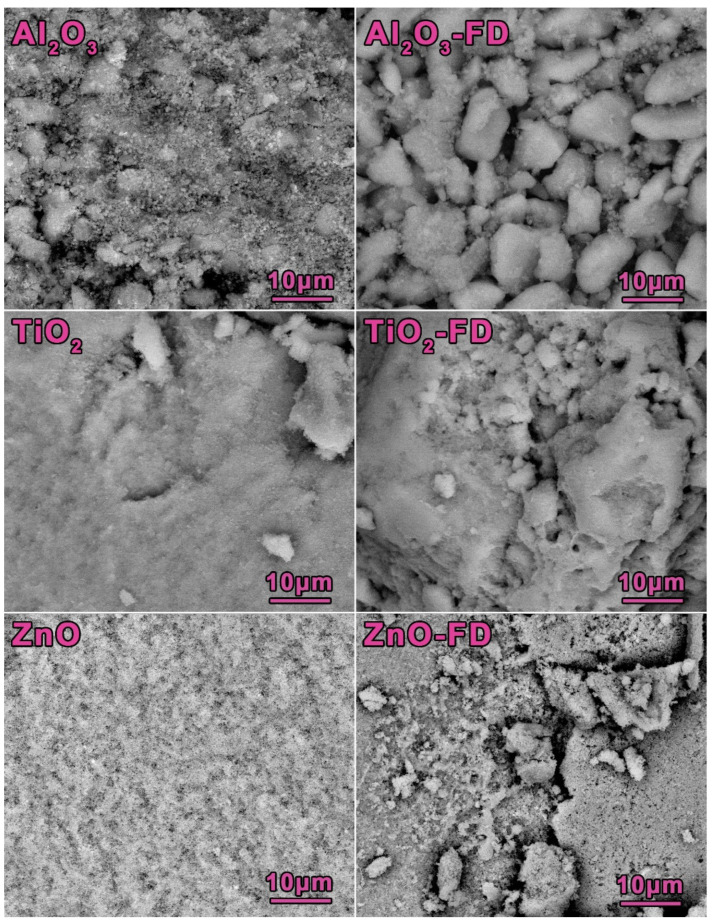
Scanning electron micrographs of the pure oxides and fucoidan-oxide hybrid materials, magnification 2500×.

The carbon and hydrogen elements present in the pure oxides come from the adsorbed water and carbon dioxide (which are present in the Earth atmosphere), whereas the increase of these elements in the hybrid materials originates from the adsorbed fucoidan. Figure 2 shows the surface morphology of the studied oxides and fucoidan-oxide hybrid materials. It can be observed that the materials containing the polymer form larger aggregates, characterized by a more non-uniform structure.

Simultaneous thermogravimetric (TGA) and calorimetric (DSC) analyses of pure fucoidan, metal oxides and the obtained hybrid materials were performed to determine their thermal stability and decomposition paths in the air.

**Figure 3:**
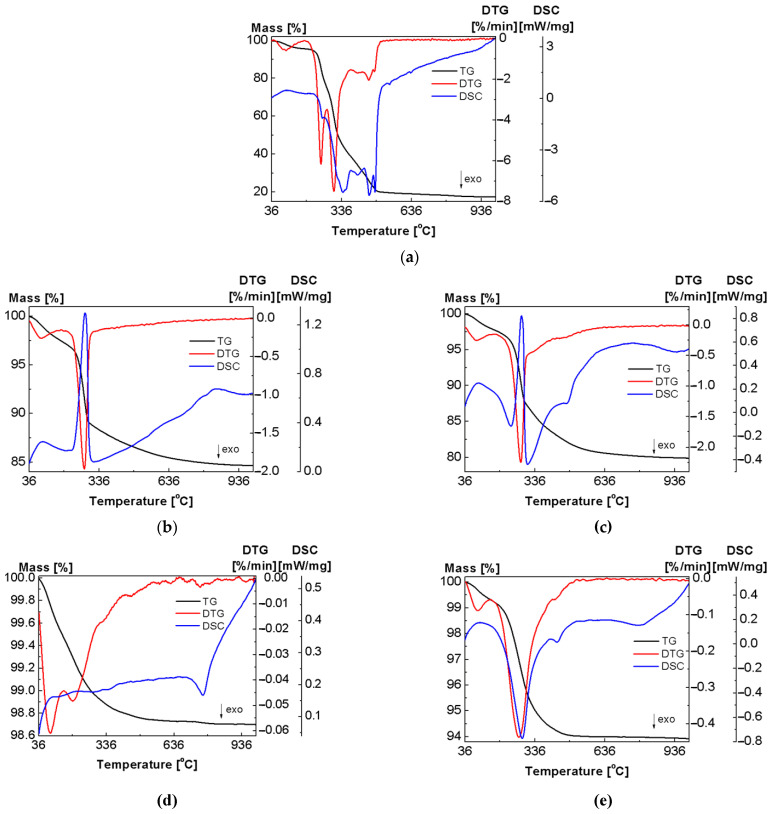

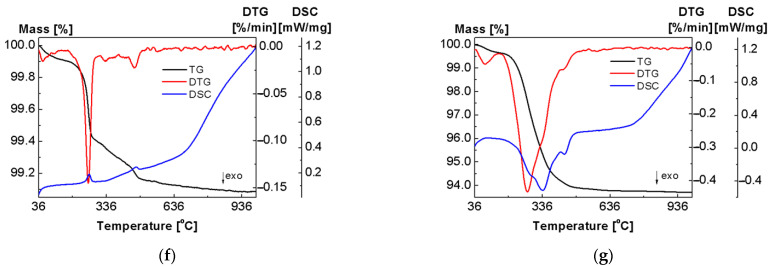
TG/DTG-DSC curves for: (**a**) FD (5.17 mg), (**b**) Al_2_O_3_ (20.00 mg), (**c**) Al_2_O_3_-FD (20.02 mg), (**d**) TiO_2_ (35.02mg), (**e**) TiO_2_-FD (35.06 mg), (**f**) ZnO (35.13 mg) and (**g**) ZnO-FD (34.98 mg) in the air atmosphere.

**Figure 4:**
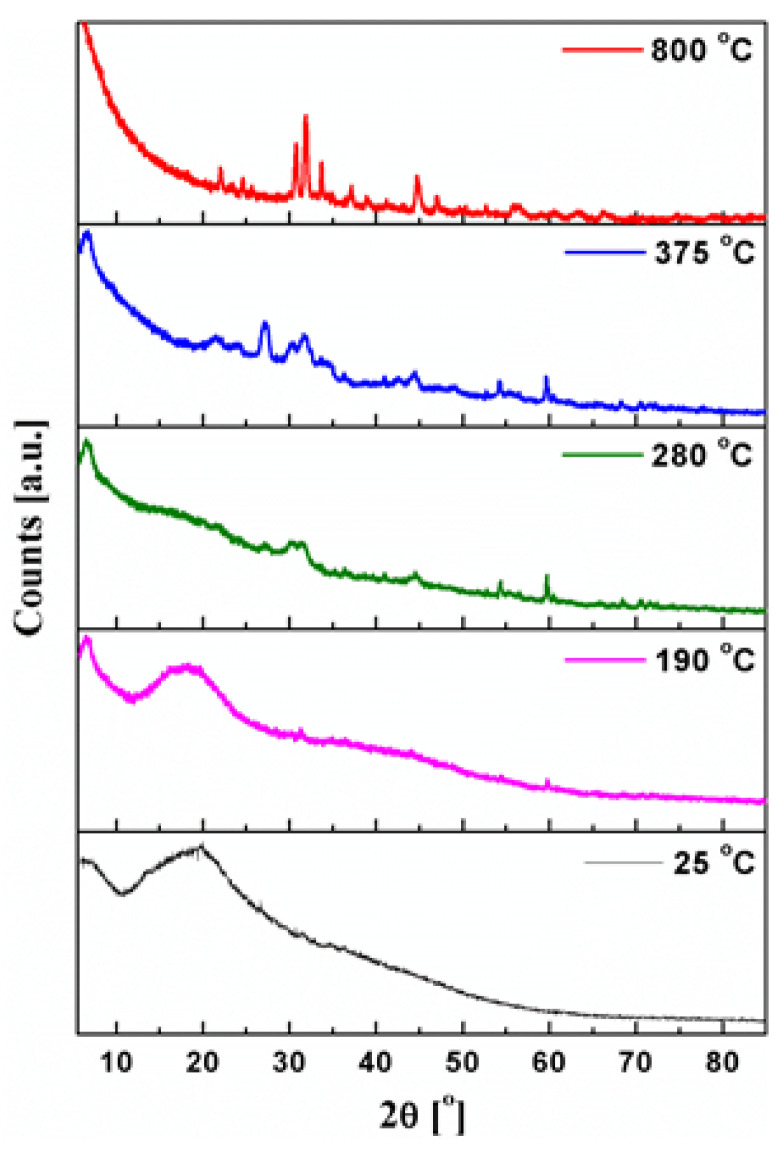
Powder XRD patterns of FD registered during the heating in the range of 25–800 °C in the air.

The TGA curves shown in Figure 3a indicate that the decomposition of fucoidan takes place in four stages. The first stage is the removal of physically absorbed water, but the second step is associated with the simultaneous removal of chemisorbed water and the onset of the fucoidan molecule degradation. The third and fourth stages are the continuation of the polysaccharide macromolecule degradation [25,26]. The first decomposition step occurs in the range of 36–200 °C and corresponds to a moisture loss of 4.38%. This effect exhibits a single endothermic transition at a peak temperature (T_peak_) of 97 °C which is visible on the DSC thermogram. The temperature diffractograms at 25–190 °C (Figure 4) show that the structure of the native polysaccharide is amorphous. The removal of physically adsorbed water has a slight influence on the XRPD patterns, and the intermediate anhydrous product is still amorphous.

According to the DTG profile, further thermal degradation of fucoidan proceeds in three overlapping stages and is accompanied by several strong exothermic processes as can be observed on the DSC curve and the data presented in Table 3. The second and third steps of the thermal degradation represent the minor and major devolatilisation processes, respectively. The second phase starts at 200 °C and ends at 275 °C with a mass loss of 20.57%, while the weight loss at the end of the third phase at 375 °C is 60.83%. The maximum speed of the decomposition process (T_max_) observed at 249 °C and 305 °C (Table 3) represents the release of structural water bound to FD and the release of molecular products from the thermal degradation of the biopolymer. The last phase of oxidative degradation starts at 375 °C and finishes at 1000 °C with a mass loss of 21.47% (T_max_ value at 454 °C and 479 °C, respectively). Thus, the total weight loss of pure FD at the end of the fourth stage exceeds 82%.

**Figure 5:**
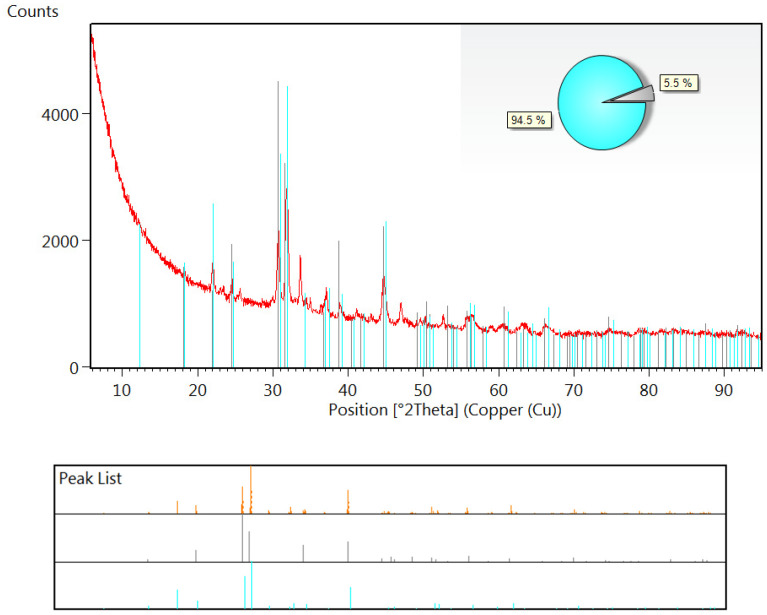
Identification of the decomposition products of FD at 800 °C in the air. The presence of potassium sodium sulfate (Ref. Code 04-009-3429—KNa(SO_4_)—light blue in the chart) and potassium carbonate (04-013-9892—K_2_CO_3_—grey in the chart) was proved using the ICDD PDF4+2021 diffraction database.

The X-ray diffraction patterns at 280 and 375 °C (Figure 4) show that the stepwise oxidation of organic matter and/or gaseous products results in increased crystallinity of solid residues. In the air atmosphere, the thermal degradation of fucoidan is practically completed up to 600 °C (Figure 3a) with the total mass loss equal to 82.3% and the degradation of the final crystalline product (Figure 4). Its identification revealed that the solid product of the thermal degradation of fucoidan in the air is a mixture of potassium sodium sulfate and potassium carbonate (Tables S1 and S2). Moreover, the Rietveld method established that the double salt is dominant in this mixture (Figure 5).

**Figure 6:**
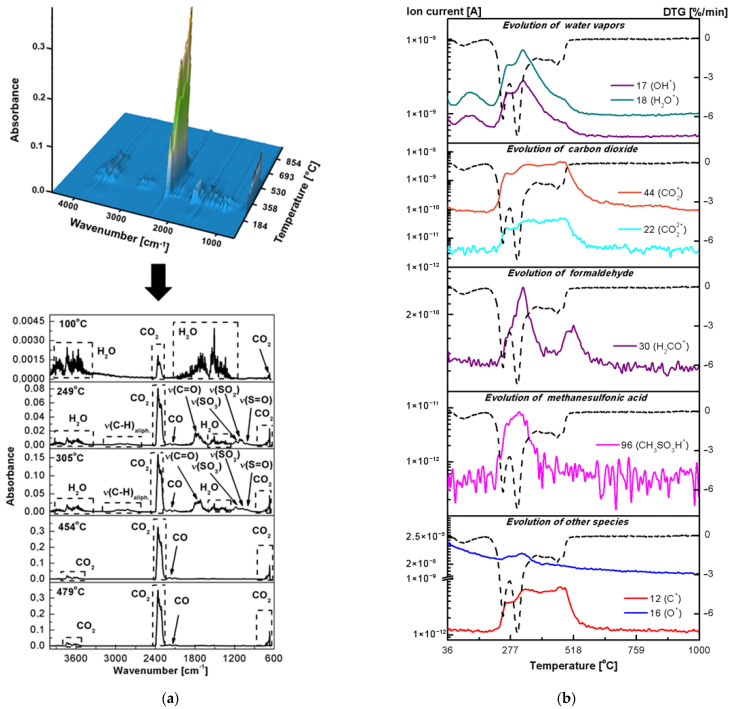
Results of the EGA analysis in the air for FD: (**a**) 3D-2D FTIR spectra of gas products, (**b**) the selected ionic masses of gaseous products and the corresponding ionic current tracks (the solid line) and a DTG run (the dotted line).

To identify the unknown gaseous products of the thermal decomposition of fucoidan, oxides, and fucoidan-oxide hybrid materials and to confirm their postulated degradation paths in the air atmosphere, the evolved gas analysis (EGA) was performed, applying the simultaneous TG-FTIR and TG-MS techniques. The FTIR spectra of the gases evolved at the specific temperatures for fucoidan are shown in Figure 6a. The ion currents for the selected fragments with the corresponding DTG curve as a function of temperature are presented in Figure 6b.

In the FT-IR spectrum of fucoidan at 100 °C (Figure 6a), characteristic bands were observed in the ranges of 3550–3200, 1630–1600 and 600–400 cm^−1^ corresponding to the asymmetric and symmetric OH stretching, HOH bending and the rotational oscillations of the water molecules [27]. Simultaneously, the ionic current scans in the mass spectra at the temperature of 25–200 °C showed the signals at *m*/*z* 17 and 18 (Figure 6b) related to the OH^+^ and H_2_O^+^ species [28,29]. Furthermore, no other MS signals and absorption bands from the small molecules were observed in the temperature range of 36–200 °C, indicating that the fucoidan chains are not destroyed. Thus, based on the results of the TG–FTIR–MS analysis, it was confirmed that the first stage of the thermal degradation of the polysaccharide corresponds only to the water removal. As postulated earlier, the second stage of the thermal degradation of fucoidan corresponds to the simultaneous removal of structural water and the onset of oxidative decomposition of the fucoidan structure. In addition to the previously described FTIR bands and the MS signals characteristic of water vapour evolution, some small CO and CO_2_ molecules, as well as the gaseous products related to the organic molecules (fragments of monosaccharide units) were also detected. The presence of carbon dioxide is confirmed by a characteristic doublet in the range of 2400–2250 cm^−1^ and 750–600 cm^−1^ attributed, respectively, to the asymmetric and bending stretching vibrations of the molecule in the IR spectrum at 249 °C (Figure 6a). The ion current tracks corresponding to the *m*/*z* 22 (CO_2_^2+^) and 44 (CO_2_^+^) ions in the mass spectrum in the temperature range 200–249 °C are shown in Figure 6b. The weak bands at 2179 and 2102 cm^–1^ in the IR spectrum at 249 °C confirmed carbon monoxide release [28]. In addition, the EGA results showed some interesting information on the gaseous organic molecular fragments emitted during the fucoidan degradation above 200 °C. In the FTIR spectrum at 249 °C, the bands including the characteristic vibrations ν_C-H_ (2840–3070 cm^−1^) of the alkyl, ν_C=O_ (1800–1700 cm^−1^) of the carbonyl (aldehyde), as well as ν_S=O_, ν_SO2_ and ν_SO3_ (1200–1000 cm^−1^) of the sulfate ester groups were identified [27]. Comparing the experimental spectra with the databases and the literature data [30,31], it can be concluded that the destruction of the polysaccharide structure during heating in the air atmosphere results in the formation of formaldehyde and methanesulfonic acid as gaseous products. This fact was confirmed by the registered mass spectra in which the signals with *m*/*z* 30 and 96 from the molecular ions: H_2_CO^+^ [32,33,34] and CH_3_SO_3_H^+^ [35] were detected (Figure 6b). Furthermore, in the mass spectra, far more signals were observed confirming the evolution and cleavage of formaldehyde (Figure S1) and methanesulfonic acid (Figure S2). It must be noted that one of the major fragment ions that is formed during the formaldehyde cleavage is CO^+^ (*m*/*z* 28). This explains the presence of carbon monoxide in the gaseous products of the fucoidan thermal degradation. On the other hand, the presence of ions of the aliphatic groups CH_3_^+^, CH_2_^+^, and CH^+^ indicates the defragmentation of monosaccharide rings. In the third degradation stage of fucoidan, the composition of the evolved gases was the same as in the second one. Thus, in the temperature range of 275–375 °C, water vapours, carbon oxide, carbon dioxide, formaldehyde and methanesulfonic acid were detected (Figure 6a,b). From the EGA results, it was known that the fourth decomposition step of the polysaccharide takes place with the emission of formaldehyde and CO as well as CO_2_ and H_2_O, which are attributed to the combustion of the organic matter. In the FTIR spectra at 454 and 479 °C, only the characteristic bands of carbon oxides were visible (Figure 6a). However, in the mass spectra, the signals with *m*/*z* of 17 and 18 corresponding to water as well as with *m*/*z* 30 related to the molecular ion H_2_CO^+^ were detected (Figure 6b).

The TGA–DSC curves of the Al_2_O_3_, TiO_2_ and ZnO oxides were recorded at temperatures between 36–1000 °C to confirm their purity and thermal stability. The TGA plots indicate the total weight loss for the studied oxides equal 15.35, 1.3 and 0.91%, respectively (Figure 3b,d,f). The initial stage is between room temperature and 150 °C, which is due to the loss of physically adsorbed water. The second stage started at 150 °C and ended at ~500 °C due to the loss of chemisorbed water [36,37,38]. On the corresponding DSC curves, the dehydration of these oxides is accompanied by a very weak endothermic effect (ZnO, TiO_2_) and a strong endothermic effect (Al_2_O_3_), consistent with the increase in the degree of their surface hydroxylation. There is a negligible weight loss at the temperatures above 500 °C. However, on further heating, some phase transitions are observed on the TiO_2_ and Al_2_O_3_ DSC thermograms. The exothermic peaks represent the crystallization processes whereas the endothermic ones are associated with the dissolution of the crystalline phases. Titania (TiO_2_) is known to exist in nature as three major polymorphs: anatase, brookite, and rutile. Rutile is the only stable phase of microsized TiO_2_, whereas anatase and brookite are metastable at all temperatures and convert to rutile when heated [39]. The DSC curve of the TiO_2_ sample shows a sharp exothermic peak at 760 °C due to the slow phase conversion from the anatase phase to the rutile phase [40]. Upon heating, metastable γ-Al_2_O_3_ undergoes a series of polymorphic phase transitions to more ordered cubic closely packed θ-Al_2_O_3_, which heated to ~1200 °C undergoes a transformation to form thermodynamically stable α-Al_2_O_3_ [37,41]. On the DSC thermogram of Al_2_O_3_ (Figure 3b), a wide range of endothermic peak is observed due to the conversion of γ-Al_2_O_3_ into θ-Al_2_O_3_ at ~840 °C [42]. Figures S3a, S4a and S5a show the results of the EGA-FTIR analysis of Al_2_O_3_, TiO_2_ and ZnO in the air atmosphere as stacked plots of the FTIR spectra of the gaseous species released as selected. Moreover, Figures S3b, S4b and S5b show the EGA-MS curves as the ion current changes of the specific mass fragments of the gaseous species evolved in comparison with the DTG profiles versus temperature. As expected, all investigated metal oxides released a very small amount of gas during heating in the temperature range 36–1000 °C. Based on the EGA analysis, it was confirmed that the slight mass losses were due to the dehydration process. Therefore, in the IR spectra (Figures S3a, S4a and S5a) at T_max_, characteristic bands in the ranges 3550–3200, 1630–1600, and 600–400 cm^−1^ were observed, corresponding to the asymmetric and symmetric O-H stretching, HOH bending and the rotational vibrations of water molecules, respectively. Simultaneously, in the mass spectra (Figures S3b, S4b and S5b), the signals at *m*/*z* 17 and 18 attributed to the OH^+^ and H_2_O^+^ species were detected. In addition, the evolution of carbon dioxide adsorbed on the oxide surface was evidenced [27,28,29].

**Figure 7:**
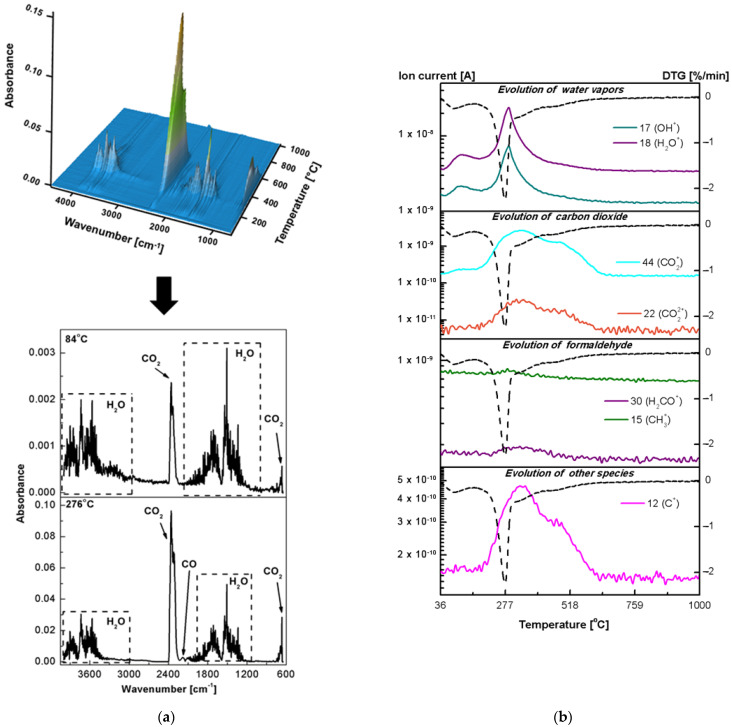
Results of the EGA analysis in the air for the Al_2_O_3_-FD: (**a**) 3D-2D FTIR spectra of the gas products, (**b**) the ionic masses of gaseous products and the corresponding ionic current tracks (the solid line) and the DTG run (the dotted line).

**Figure 8:**
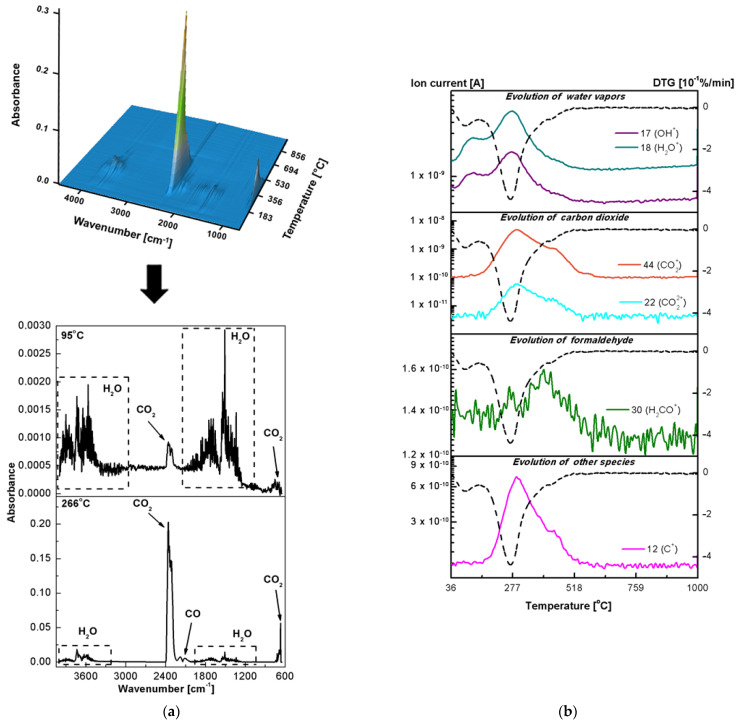
Results of the EGA analysis in the air for the TiO_2_-FD: (**a**) 3D-2D FTIR spectra of the gas products, (**b**) the ionic masses of the gaseous products and the corresponding ionic current tracks (the solid line) and the DTG course (the dotted line).

**Figure 9:**
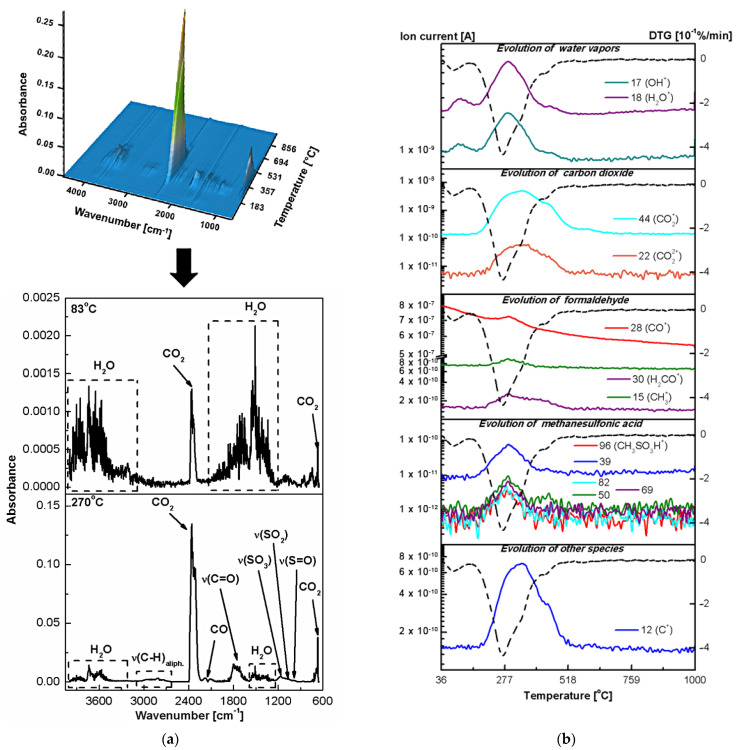
Results of the EGA analysis in the air for the ZnO-FD: (**a**) 3D-2D FTIR spectra of the gas products, (**b**) the ionic masses of gaseous products and the corresponding ionic current tracks (the solid line) and the DTG run (the dotted line).

The thermal properties and behaviour are important material parameters. Therefore, both the classic thermogravimetric and the advanced thermal analysis were performed in the simulated airflow to investigate purity and stability of the newly obtained hybrid materials. Figure 3b–g shows the TG-DTG-DSC curves of the pure oxides and fucoidan-oxide hybrid materials. Although the percentage of adsorbed fucoidan in the hybrid materials is not significant, the decomposition paths of the Al_2_O_3_-FD, TiO_2_-FD and ZnO-FD systems are different from those presented by fucoidan and the pure oxides. The destruction curves obtained for the hybrid materials result from the effects observed during the thermal analysis of their pure components. The comparison of the DTG curves in Figure 1 shows that the degradation processes of the hybrid materials take place in two stages, while the thermal decomposition of fucoidan is a four-stage process. It is also worth emphasizing that the thermal stability of fucoidan also increased in the presence of the oxides, indicating the interactions between the biopolymer and the oxides. The first Al_2_O_3_-FD decomposition step occurs in the range of 25–175 °C and corresponds to the dehydration process with a mass loss of 2.40%. The dehydration is associated with the endothermic effect at 93 °C on the DSC curve. Further heating leads to the next decomposition stage with a broad peak in the DTG centred at 276 °C (Figure 3c, Table 3). This stage is related to the simultaneous dehydration and degradation of the adsorbed fucoidan. The endotherm centred at T_peak_ of 279 °C (related to the dehydration) and two exothermic peaks centred at 305 and 475 °C (related to the oxidative decomposition of fucoidan) confirm these effects clearly. It should be noted that in the form of the Al_2_O_3_-FD hybrid material, fucoidan exhibits a greater thermal stability because its decomposition occurs at a higher temperature (T_max_ = 276 °C, T_peak_ = 279 °C) than in the pure form (T_max_ = 249 °C, T_peak_ = 255 °C). The total mass loss in the thermal degradation of the Al_2_O_3_-FD is 20.09%, while 15.36% was found in the case of pure oxide. The thermal degradation of the TiO_2_-FD also proceeds in two stages (Figure 3e, Table 3). There is a slight decrease in weight (0.69%) at 36–150 °C associated with the water loss. Contrary to the results obtained for the Al_2_O_3_-FD, the analysis of the DSC course in this range revealed that there is no distinct endothermic peak assigned to the dehydration step. This may be caused by the fact that the mass loss is small and occurs slowly, so the heat involved in this step is not sufficient to cause such a thermal event [43]. Similarly to the Al_2_O_3_-FD system, the dehydrated TiO_2_-FD does not exhibit a considerable thermal stability, and the additional heating causes the next degradation step that corresponds to the extended peak on the DTG curve with T_max_ at 266 °C and a total weight loss of 5.38% as well as the three wide exothermic peaks related to T_peak_ of 282, 430 and 780 °C. It can be assumed that the weight loss in the temperature range of 150–500 °C is due to the degradation and oxidation of the organic matter and/or gaseous products evolved during the thermal decomposition of adsorbed fucoidan. The comparison of the TG-DTG/DSC curves of pure fucoidan and the TiO_2_-FD also revealed that fucoidan in the hybrid material exhibits a greater thermal stability because its decomposition occurs at a higher temperature on the DTG and DSC pattern (T_max_ = 266 °C, T_peak_ = 282 °C) than in pure form (T_max_ = 249 °C, T_peak_ = 255 °C). Furthermore, the broad exotherm with T_peak_ at about 780 °C shows that the phase transition from anatase to rutile occurs much more slowly and at higher temperatures than in pure TiO_2_ (760 °C). The thermal analysis of the ZnO-FD system (Figure 3g, Table 3) indicates that its behaviour is similar to that of the materials described above, especially to the TiO_2_-FD system; therefore, its degradation is also a two-stage process. The first step that corresponds to the moisture removal occurs at 25–150 °C with the T_max_ value peak at 83 °C on the DTG curve and a slight mass loss of 0.34%, so there is only a very small endothermic effect and a wide DSC curve that corresponds to this dehydration process. This is probably due to too little weight loss to trigger a thermal event. The next decomposition step occurs at 150–550 °C. In this range, the DTG curve displays a broad peak with the maximum speed of decomposition at 270 °C as well as the exothermic peaks in the DSC course at 336 and 433 °C are present. The observed mass loss (5.93%) is related to the oxidative decomposition of the organic matter originating from fucoidan. As for the previously described hybrid materials, the ZnO-FD degradation of adsorbed fucoidan also proceeds at a higher temperature than that observed for the pure compounds. The 3D and 2D plots of the infrared spectra extracted at the different temperatures of the gaseous products evolved during the thermal decomposition of the fucoidan-oxide hybrid materials in the air are presented in Figure 7, Figure 8 and Figure 9a. The profiles of the ion currents of the identified mass fragments of the gaseous species evolved versus temperature as well as the corresponding DTG curves are shown in Figure 7b, Figure 8b and Figure 9b.

The EGA analysis confirmed that of the two-stage thermal degradation processes of the newly obtained hybrid materials, the first one was due to the dehydration. For each oxide-FD hybrid material in the IR spectrum of the gaseous products evolved below 200 °C (Figure 7a, Figure 8a and Figure 9a), the presence of the characteristic water absorption bands (3550–3200, 1630–1600 and 600–400 cm^−1^) was observed. Furthermore, in the temperature range 36–200 °C, the signals with *m*/*z* 17 and 18 corresponding to the OH^+^ and H_2_O^+^ species (Figure 7b, Figure 8b and Figure 9b) were detected. The prediction based on the simultaneous TG-DTG-DSC curves of the second step of the thermal decomposition of the Al_2_O_3_-FD, TiO_2_-FD and ZnO-FD was also confirmed. In addition to the bands and signals related to water evaporation, in each case, the products of the thermal degradation of fucoidan were found (Figure 7, Figure 8 and Figure 9). For the Al_2_O_3_-FD and the TiO_2_-FD hybrid materials, the evolution of the gas during the second decomposition step was not significant. However, significant absorption bands of carbon monoxide (Figure 7a and Figure 8a) as well as weak signals corresponding to the molecular ion of formaldehyde (Figure 7b and Figure 8b) were detected. On the other hand, the analysis of the gaseous products evolved during the degradation of the ZnO-FD system proved the presence of both formaldehyde and its derivative, carbon monoxide, as well as methanesulfonic acid (Figure 9).

## 3. Materials and Methods

### 3.1. Materials

In the following studies, three different commercially available nanosized oxides were used: aluminium(III) oxide (gamma form), titanium(IV) oxide (anatase form) and zinc(II) oxide (zincite form). The properties of the oxides were previously discussed [44]. The above-mentioned oxides were supplied from AlfaAesar/ThermoFisher Scientific (Kendal, Germany). Before the studies, the oxides were thoroughly washed with ultrapure water to achieve the conductivity of the supernatant of 2 μS cm^−1^. Then, they were dried and kept in the desiccator to avoid water uptake. Fucoidan characterized by the molecular weight of 1,730,000.00 Da and the sulfate content estimated at 5.96% was purchased from Carbosynth Limited (Compton, United Kingdom) [24]. The polymer was used without further purification. The stock solution of fucoidan with a concentration of 3000 ppm was prepared by dissolution of 3 g of the polymer in ultrapure water. The solution was mixed using the magnetic stirrer for a few hours and then left overnight in the thermostated laboratory shaker (temperature 40 °C). The fucoidan stock solution was kept in the refrigerator (3 °C) and used within one week to avoid microbial degradation. The other reagents, sodium chloride, sodium hydroxide, sulphuric(VI) acid and hydrochloric acid, were purchased from Avantor Performance Materials Poland S.A. (Gliwice, Poland).

### 3.2. Methods

#### 3.2.1. Materials Preparation

The fucoidan-oxide hybrid materials were prepared by adsorption of the polymer from the aqueous solutions. The solution was prepared as follows: to 6.67 cm^3^ of fucoidan stock solution (3000 ppm), 1 cm^3^ of sodium chloride solution (0.01 mol dm^−3^) and 2.33 cm^3^ of ultrapure water were added. Then, 0.1 g of nano-oxide powders was added. The suspensions prepared in such way were then placed in a thermostatic bath with linear shaking for 24 h. After that, the suspensions were centrifuged to separate solids from liquids (12,000 rpm for 15 min). The obtained oxide-hybrid materials were dried in an oven at a temperature of 50 °C until mass change no longer occurred. They were kept in the desiccator for further studies.

#### 3.2.2. Materials Characterization

The obtained oxide-hybrid materials were characterized using low temperature adsorption/desorption of nitrogen (ASAP 2420, Micrometrics, Norcross, GA, USA) as well as the scanning electron microscopy (SEM, Quanta 3D FEG, Fei, Hillsboro, OR, USA). The backscattered electron detector micrographs were taken with the magnification of 2500×. The hydrodynamic diameters (Dh) of the materials were obtained using dynamic light scattering (DLS, Zetasizer NanoZS, Malvern, Worcestershire, UK). The additional elemental analysis (CHS) was also performed (EuroEA3000 CHNS-O Analyser, EuroVecor, Pavia, Italy).

#### 3.2.3. Thermal Analysis

Thermal properties were tested using a Netzsch STA 449 Jupiter F1 (Erich NETZSCH GmbH & Co. Holding KG, Selb, Germany) analyzer coupled with a Bruker Tensor FT-IR spectrometer (Bruker Corp., Billerica, MA, USA) and a Netzsch QMS 403D Aëolos mass spectrometer (Erich NETZSCH GmbH & Co. Holding KG, Selb, Germany). The samples were weighed amounting to about 5–35 mg and analyzed in the corundum crucibles in the synthetic air flow (50 mL min^−1^) in the temperature range of 36–1000 °C with a heating rate of 10 °C min^−1^. An empty alumina crucible was used as a reference. The composition of the gaseous degradation products of fucoidan, oxides and hybrid materials was studied by FTIR spectroscopy at 4000–600 cm^−1^ and mass spectrometry in the mass range of 10–100 amu. The data were collected and edited using the NETZSCH Proteus^®^ ver. 6.1 software (Erich NETZSCH GmbH & Co. Holding KG, Selb, Germany).

#### 3.2.4. X-ray Diffraction Analysis

The analysis of the crystalline phases was made using the VT XRPD method. The diffraction data were obtained by the Empyrean diffractometer with the PIXcel3D detector (PANalytical, Almelo, The Netherlands) using monochromated Cu-Kα radiation (λ = 1.54184 Å) in the 2θ range of 4.7–90°, with a step of 0.026°. The heating temperature was controlled in an XRK 900 reactor chamber (Anton Paar, Graz, Austria). The samples were heated dynamically in air and helium atmosphere with a heating rate of 10 °C min^−1^ in the range of 25–800 °C. The ICDD PDF4 + 2021 diffraction data base was used to identify crystalline phases.

## 4. Conclusions

The conducted research shows that the interactions between the studied nano-oxides and fucoidan result in the formation of hybrid materials with different properties than the starting materials. The studied textural properties of the NPs changed after functionalization by the polymer. In such a case, the hydrodynamic diameter of the studied hybrid materials increased in comparison to the pure oxides. The greatest increase was observed in the case of the fucoidan-Al_2_O_3_ hybrid material. The elemental analysis confirmed the changes in the chemical composition of the studied materials. Based on the thermal analysis of fucoidan itself, it was proved that the decomposition of the polymer takes place in four stages. It was shown that fucoidan below 200 °C is prone to a loss of mass only due to dehydration. This conclusion is specifically important because of the common opinion regarding the fact that the polysaccharides are not very thermally stable. In the case of hybrid materials used in cosmetic, pharmaceutical and environmental applications, the possibility of their use at temperatures up to 200 °C opens new prospects for the final users. It was also proved that the products of thermal decomposition of amorphous fucoidan, potassium sodium sulfate and potassium carbonate, are crystalline. The evolved gas analysis showed that formaldehyde and methanesulfonic acid are the gaseous products of the thermal decomposition of fucoidan. As far as the thermal properties of the hybrid materials are concerned mixing together contributed to the increase of thermal stability. Using both the conventional thermogravimetric and advanced thermal analysis, the decomposition paths of the Al_2_O_3_-FD, TiO_2_-FD and ZnO-FD systems were proved to be different from those presented by fucoidan and pure oxides. The degradation processes of the hybrid materials take place in two stages, but the thermal decomposition of fucoidan is a four-stage process.

The obtained data show that the Al_2_O_3_-FD, TiO_2_-FD and ZnO-FD hybrid materials, prepared by functionalization of the NPs by fucoidan, are characterised by different thermal properties than the pure components. Because of the well-known bioactive properties of fucoidan, such materials can be of significant importance in the cosmetic and pharmaceutical industries. The next step in the presented studies could be the investigation of the in vivo and in vitro interactions between such materials and the living organism. Furthermore, based on the literature data, it could be assumed that the fucoidan-oxide hybrid materials are characterized by greater antioxidant properties which might be helpful in inflammation and skin diseases treatment. However, to support such hypotheses, further studies are required. Moreover, such a finding could help industrial formulators in the preparation of new products appreciated by customers. Therefore, the presented study not only fills the void regarding the preparation of the fucoidan-oxide hybrid materials but is also characterized by great application potential.

## Figures and Tables

**Table 1:** Hydrodynamic diameters (D_h_) of the oxides and the fucoidan-oxide hybrid materials measured in the ultrapure water.

	Al_2_O_3_	Al_2_O_3_-FD	TiO_2_	TiO_2_-FD	ZnO	ZnO-FD
Hydrodynamic diameter, Z-average [nm]	344.8 ± 16.5	676.1 ± 42.9	173.8 ± 15.6	225.1 ± 2.7	289.8 ± 26.6	322.60 ± 11.6
Polydispersity index, PDI	0.226	0.399	0.224	0.250	0.239	0.298

**Table 2:** Surface properties of pure oxides and hybrid materials obtained from the nitrogen adsorption-desorption isotherms at −196 °C.

Parameter	Value					
	Al_2_O_3_	Al_2_O_3_-FD	TiO_2_	TiO_2_-FD	ZnO	ZnO-FD
BET specific surface area [m^2^ g^−1^]	171.3	134.2	50.3	47.5	13.6	9.8
Micropore area [m^2^ g^−1^]	20.17	11.37	3.97	2.89	2.78	1.94
External surface area [m^2^ g^−1^]	151.16	122.87	46.35	44.59	10.76	7.87
Micropore volume [cm^3^ g^−1^]	0.007638	0.004288	0.001460	0.000936	0.001223	0.000889

**Table 3:** Thermogravimetric analysis of fucoidan, metal oxides, and hybrid materials in the air atmosphere (where: Δm—the mass loss; T_peak_—the peak temperature on the DSC curve; T_(max)_—the temperature at the maximum mass loss on the DTG curve). Thermal effects: (-) endo, (+) exo.

Stage	T_peak_ [°C]	Thermogravimetry		
		Temp. Range; T_max_, [°C]	Δm [%] Found	Residue [%] Found
FD				
I	97(-)	25–200; 100	4.38	
II	255(+)	200–275; 249	20.57	
III	343(+)	275–375; 305	35.88	
IV	406(+)	375–1000; 454, 479	21.47	17.7
	455(+)			
	482(+)			
Al_2_O_3_				
I	93(-)	25–210; 87	2.93	
II	276(-)	210–1000; 273	12.43	
	840(-)			84.64
Al_2_O_3_-FD				
I	93(-)	25–175; 84	2.40	
II	279(-)	175–1000; 276	17.69	
	305(+)			
	475(+)			
	762(-)			79.91
TiO_2_				
I	93(-)	25–150; 90	0.53	
II	192(-)	125–1000; 187	0.77	98.70
TiO_2_-FD				
I	95(-)	25–150; 95	0.69	
II	282(+)	150–1000; 266	5.38	
	430(+)			93.93
	780(+)			
ZnO				
I	70(-)	25–175; 55	0.10	
II	259(-)	175–285; 255	0.49	
III	468(-)	285–1000; 459	0.32	
				99.09
ZnO-FD				
I	95(-)	25–150; 83	0.34	
II	336(+)	150–1000; 270	5.93	
	433(+)			93.73

## Data Availability

Not applicable.

## Supplementary Materials

The following supporting information can be downloaded at: https://www.mdpi.com/article/10.3390/ijms23020805/s1.
