# Supplementary material for: Textural and Thermal Properties of the Novel Fucoidan/Nano-Oxides Hybrid Materials with Cosmetic, Pharmaceutical and Environmental Potential
Source: Int J Mol Sci. 2022 Jan 12;23(2):805. doi: 10.3390/ijms23020805 (PMC8775903; doi:10.3390/ijms23020805)
Supplement: Supplementary file 1 [file ijms-23-00805-s001.zip › Supplementary material_R1.pdf]

Supplementary material

Textural and thermal properties of the novel fucoidan/nano-oxides hybrid materials with cosmetic, pharmaceutical and environmental potential

Jakub Matusiak [1*], Urszula Maciołek [2], Małgorzata Kosińska-Pezda [3], Dariusz Sternik [4], Jolanta Orzeł [5] and Elżbieta Grządka [5,]

[1] Institute of Chemical Sciences, Faculty of Chemistry, Maria Curie-Sklodowska University, M. Curie-Sklodowska Sq. 3, 20-031 Lublin, Poland; jakub.matusiak@poczta.umcs.lublin.pl

[2] Analytical Laboratory, Institute of Chemical Sciences, Faculty of Chemistry, Maria Curie-Skłodowska University, M. Curie-Sklodowska Sq. 3, 20-031 Lublin, Poland; urszula.maciolek@mail.umcs.pl

[3] Department of Inorganic and Analytical Chemistry, Faculty of Chemistry, Rzeszow University of Technology, Rzeszow, Poland; m.kosinska@prz.edu.pl

[4] Department of Physical Chemistry, Institute of Chemical Sciences, Faculty of Chemistry, Maria Curie- Sklodowska University in Lublin, M. Curie-Sklodowska Sq. 3, 20-031, Lublin, Poland; dariusz.sternik@mail.umcs.pl

[5] Department of Radiochemistry and Environmental Chemistry, Institute of Chemical Sciences, Faculty of Chemistry, Maria Curie-Sklodowska University, M. Curie-Sklodowska Sq. 3, 20-031 Lublin, Poland; jolanta.orzel@mail.umcs.pl, elzbieta.grzadka@mail.umcs.pl

* Correspondence: jakub.matusiak@poczta.umcs.lublin.pl;

Table S1. Powder diffraction pattern for $KNa(SO_4)$ (CuKα radiation, I/Imax ≥ 5) with hkl Miller indices

2θ [°]	18.26	22.09	24.79	30.96	31.90	34.32	37.00	37.57	39.14	45.05	56.26	56.84	61.29	64.53	66.68	75.32
I/I_{max}	6.6	40.8	17.5	68.9	100.0	6.6	5.9	12.1	10.5	46.3	11.9	11.3	9.3	5.4	12.8	6.7
hkl	100	101	002	102	110	111	200	003	201	022	212	300	114	204	220	132

Table S2. Powder diffraction pattern for K_2CO_3 (CuKα radiation, I/Imax ≥ 5) with hkl Miller indices

2θ [°]	24.57	30.68	31.62	38.79	44.64	49.19	50.36	50.88	53.25	55.73	56.31	60.69	66.03	74.56	87.47
I/I_{max}	24.9	100.0	63.4	34.2	42.8	6.7	11.3	5.5	9.9	8.4	5.9	11.7	7.4	8.3	5.0
hkl	002	102	110	201	202	210	004	211	203	212	300	114	220	312	224

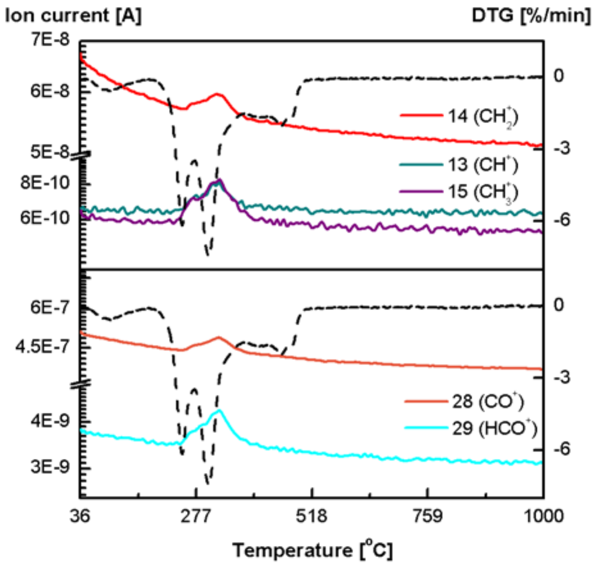

Figure S1. The ionic masses of the gaseous products of the formaldehyde cleavage and the corresponding ionic current tracks (the solid line) and a DTG run (the dotted line) from the thermal decomposition of fucoidan.

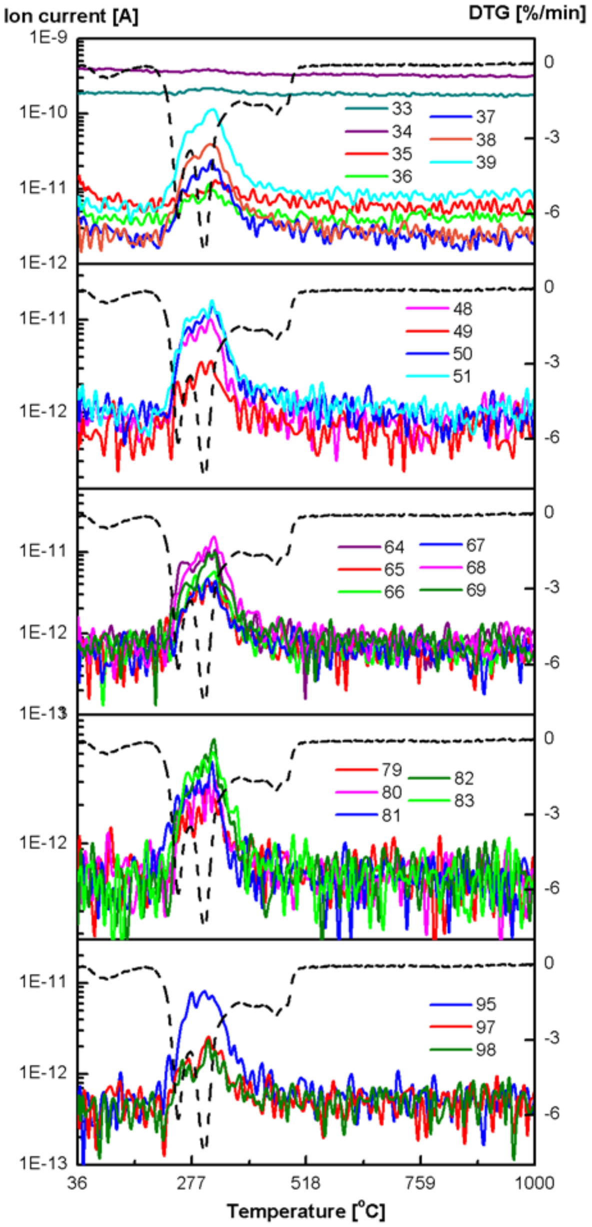

Figure S2. The ionic masses of the gaseous products of the methanesulfonic acid cleavage and the corresponding ionic current tracks (the solid line) and a DTG run (the dotted line) from the thermal decomposition of fucoidan.

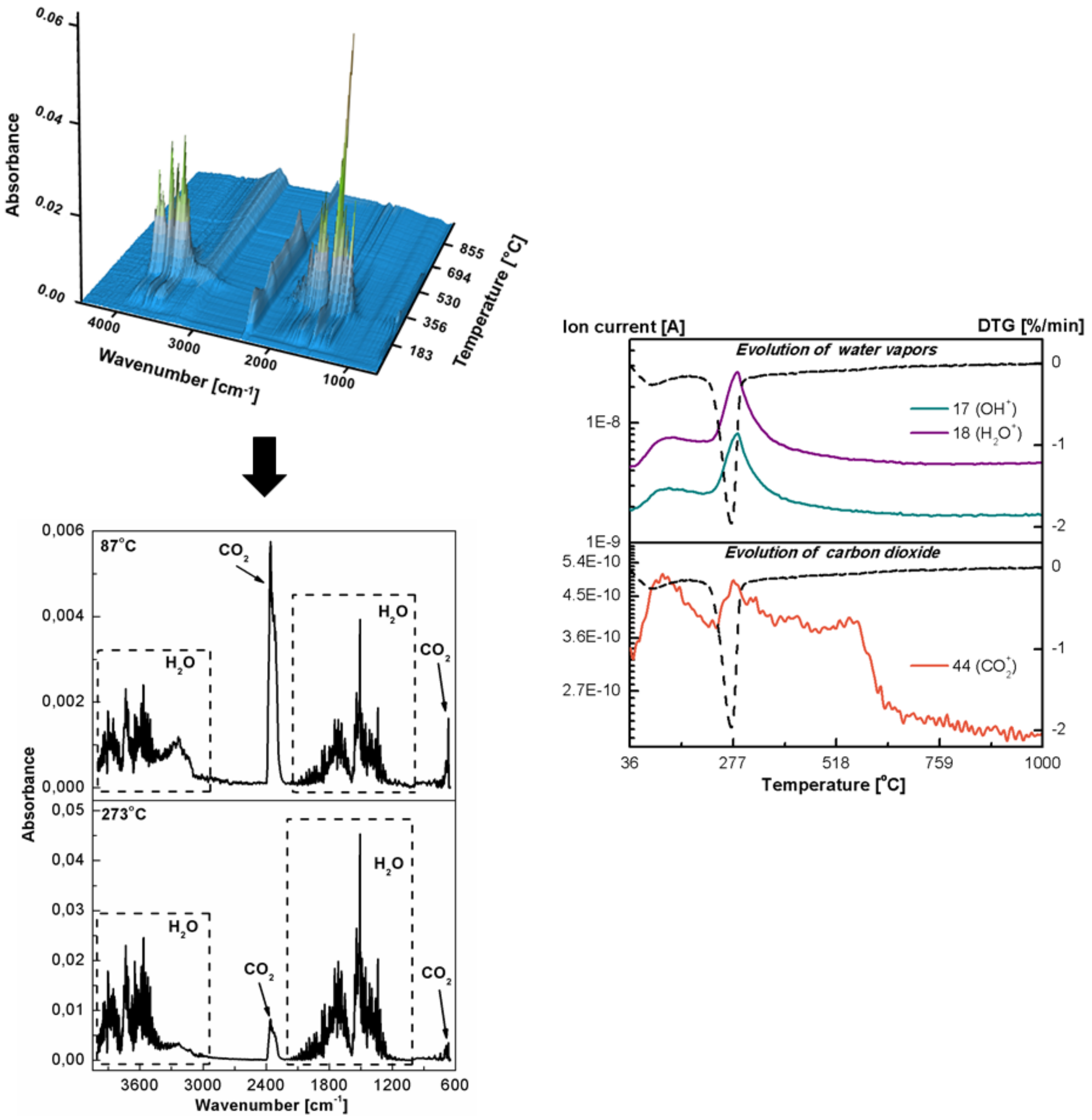

(a) **(b)**

Figure S3. Results of the EGA analysis in the air for Al_2O_3: (a) the 3D-2D FTIR spectra of the gas products (b) the ionic masses of the gaseous products and the corresponding ionic current tracks (the solid line) and a DTG run (the dotted line).

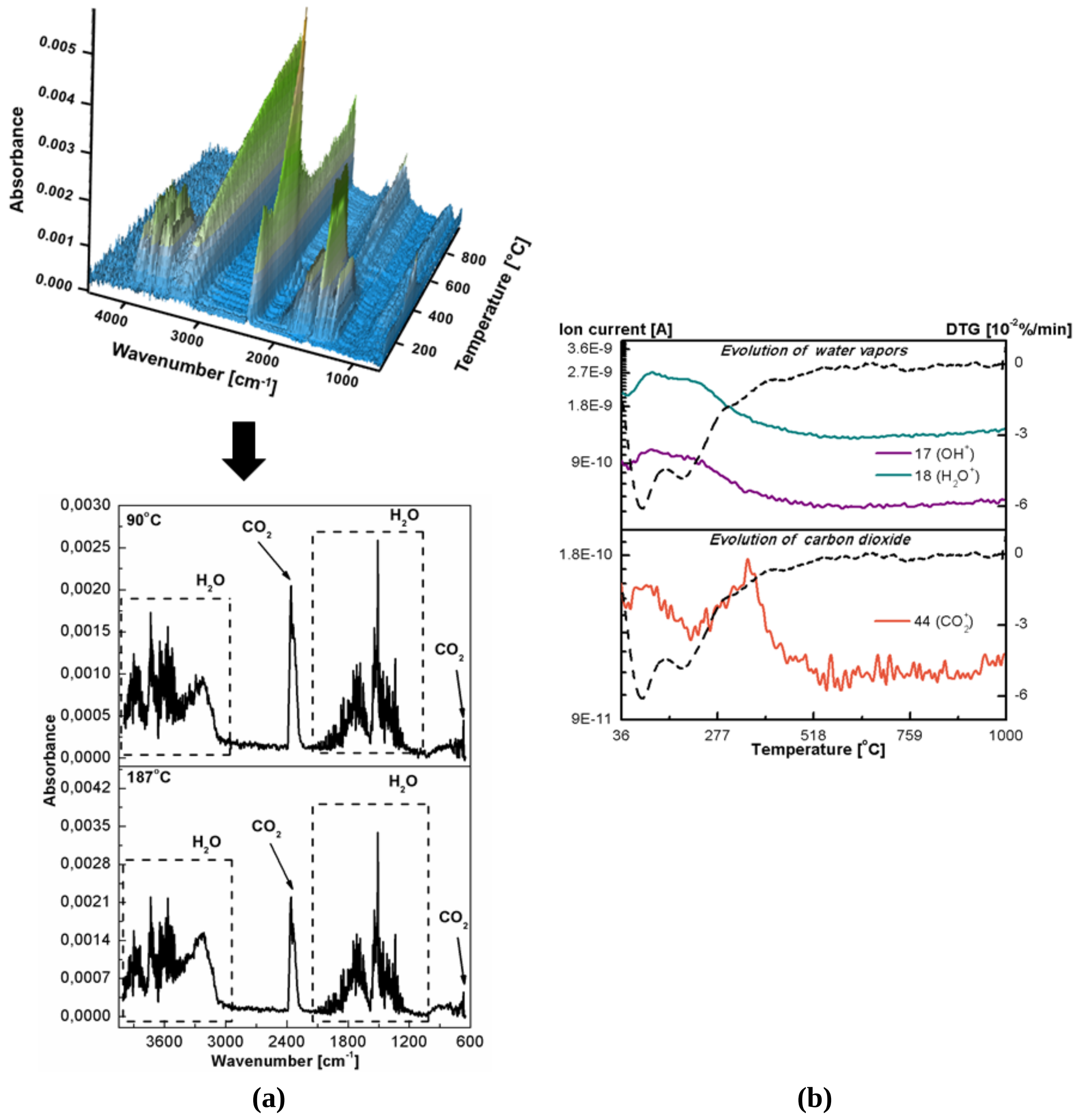

Figure S4. Results of the EGA analysis in the air for TiO_2: (a) the 3D-2D FTIR spectra of the gas products (b) the ionic masses of the gaseous products and the corresponding ionic current tracks (the solid line) and a DTG run (the dotted line).

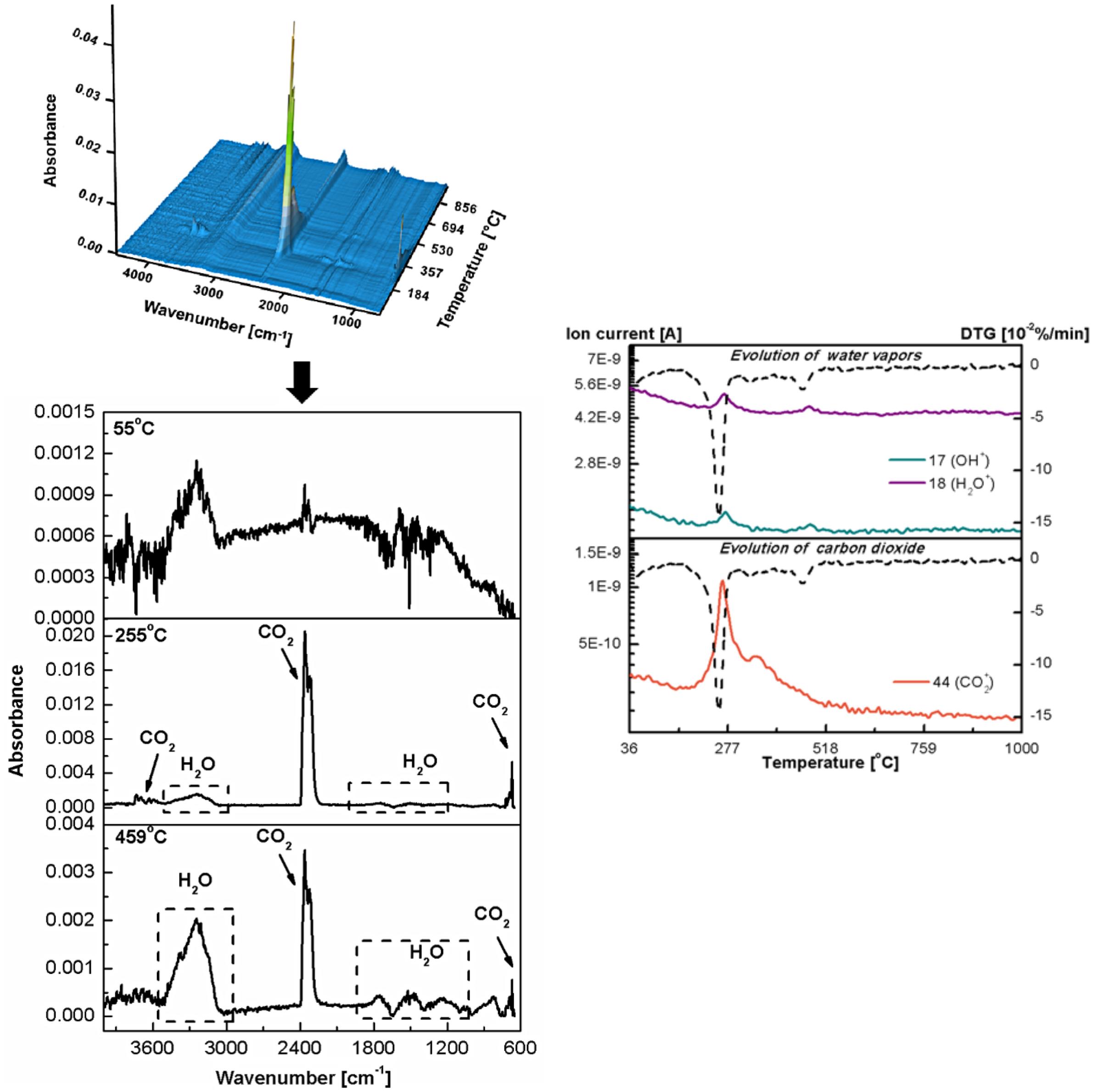

(a) **(b)**

Figure S5. Results of the EGA analysis in the air for ZnO: (a) the 3D-2D FTIR spectra of the gas products (b) the ionic masses of the gaseous products and the corresponding ionic current tracks (the solid line) and a DTG run (the dotted line).
